# Motivation and retention of health workers in developing countries: a systematic review

**DOI:** 10.1186/1472-6963-8-247

**Published:** 2008-12-04

**Authors:** Mischa Willis-Shattuck, Posy Bidwell, Steve Thomas, Laura Wyness, Duane Blaauw, Prudence Ditlopo

**Affiliations:** 1 Centre for Global Health, Trinity College Dublin, Dublin 2, Ireland; 2 Women's Health Council, Block D, Irish Life Centre, Abbey Street Lwr, Dublin 1, Ireland; 3 Centre for Health Policy, University of Witwatersrand, Johannesburg, South Africa

## Abstract

**Background:**

A key constraint to achieving the MDGs is the absence of a properly trained and motivated workforce. Loss of clinical staff from low and middle-income countries is crippling already fragile health care systems. Health worker retention is critical for health system performance and a key problem is how best to motivate and retain health workers. The authors undertook a systematic review to consolidate existing evidence on the impact of financial and non-financial incentives on motivation and retention.

**Methods:**

Four literature databases were searched together with Google Scholar and 'Human Resources for Health' on-line journal. Grey literature studies and informational papers were also captured. The inclusion criteria were: 1) article stated clear reasons for implementing specific motivations to improve health worker motivation and/or reduce medical migration, 2) the intervention recommended can be linked to motivation and 3) the study was conducted in a developing country and 4) the study used primary data.

**Results:**

Twenty articles met the inclusion criteria. They consisted of a mixture of qualitative and quantitative studies. Seven major motivational themes were identified: financial rewards, career development, continuing education, hospital infrastructure, resource availability, hospital management and recognition/appreciation. There was some evidence to suggest that the use of initiatives to improve motivation had been effective in helping retention. There is less clear evidence on the differential response of different cadres.

**Conclusion:**

While motivational factors are undoubtedly country specific, financial incentives, career development and management issues are core factors. Nevertheless, financial incentives alone are not enough to motivate health workers. It is clear that recognition is highly influential in health worker motivation and that adequate resources and appropriate infrastructure can improve morale significantly.

## Background

There is a growing need to strengthen health systems in developing countries to help meet the Millennium Development Goals (MDGs). It is widely accepted that a key constraint to achieving the MDGs is the absence of a properly trained and motivated workforce and improving the retention of health workers is critical for health system performance [1]. African countries need at least 1 million additional workers in order to offer basic services consistent with the MDGs. Instead, these countries are affected by health worker loss crippling already fragile health care systems [2]. The HIV/AIDS epidemic is compounding the problem by creating a stressful environment for health workers through increased workloads, exposure to infection and reduced morale [3].

International migration is widely blamed for the current crises and it is certainly the case that significant numbers are moving to developed countries [4]. Nurse migration has been shown to be motivated by the need for professional development, better quality of life and personal safety [5]. An estimated $500 million is spent annually on medical education of workers from Africa who will eventually emigrate [2]. In Kenya alone, US$65,997 is spent educating a single medical doctor from primary school to university and for every doctor who emigrates, US$517,931 returns in investment are lost [6].

Targeted recruitment drives for health workers from resource-poor countries have become a common solution to filling vacancies in richer countries [7]. A 'medical carousel' whereby health workers move to countries offering attractions such as better salaries and training opportunities typically leaves the poorest countries with all drain and no gain [8]. Health worker loss can compromise health system capacity to deliver adequate care as the more experienced workers migrate because their skills are highly desired. Staff shortages increase workloads and stress levels, further de-motivating remaining staff. To cope with increased workload staff are sometimes lowering their standard of care [9].

While medical migration may be dire for some countries, others intentionally export health workers in exchange for financial remittances [2]. Filipino nurses are actively exported [10,11] for which the Philippines receives over USD800 million annually [12]. There is, however, no mechanism to ensure that repatriated income will find its way into health care. Furthermore the loss of experienced personnel has a serious impact on health system progression [8]. As illustrated by a Filipino nursing director '*I am left with only novice nurses. our experienced ones go. who will teach the novice nurse? Patient complaints are frequent because our nurses are not efficient*' [9].

Health worker migration is not confined to external movement. In-country migration, from rural to urban and from public to private sector, is also creating problems with the rural areas worst affected leaving these both understaffed and the staff who are there are often under qualified [13].

Developing countries must implement strategies to protect their health systems, while recognizing health workers are autonomous people with rights. There are a growing number of studies which explore the links between incentives, motivation and retention of health workers in developing countries [14]. It is important to summarise what affects health workers to allow governments to tailor policies to alleviate the current human resource crises and a need has been identified for systemic reviews that will assist policy makers to manage human resources for health [15]. This systematic review aims to examine the importance of different motivation factors and the effectiveness of interventions to improve motivation in developing countries to reduce medical migration, both within and across countries.

## Methods

A systematic review was chosen instead of a traditional descriptive review because the use of explicit, systematic methods limits bias and reduces chance effects. The quality of the studies conducted can also be appraised and conclusions can be based on those which are methodologically most sound [16]. Accordingly, systematic review methodology was used to identify, appraise and collate evidence from studies to recognise motivational factors in developing countries as well as to describe and evaluate any interventions that have been put in place to increase health worker motivation and retention.

Motivation can be defined as 'an individual's degree of willingness to exert and maintain effort towards organizational goals' [17]. To date, results of studies on health worker motivation in developing countries have not yet been formally compared to establish common themes. A literature review conducted collecting evidence of motivation of health workers from both developing and developed contexts concluded that theories developed in western countries need be thoroughly assessed before using in a developing context [18].

This review will therefore assimilate any information in order that conclusions can be made on motivational influences affecting health workers in developing countries and to determine whether one motivational factor is more significant than others.

### Identification of data sources

Using key words the following literature databases were searched: PubMed, ISI Web of Science, Embase/Medline, Google Scholar as well as the BioMed Central 'Human Resources for Health' on-line Journal. The list of key words were: 'motivation', 'incentive', 'brain drain', 'medical migration', 'health worker migration', 'health professional', 'nurses', 'doctors', 'retention', 'policy', 'worker satisfaction' and 'developing countries'. The limitations applied to the search were 'human not animal' and 'published between 1980 and September 2007'. Language limitations were imposed, due to resource constraints and studies were only considered if an abstract and full article existed in English.

A snowball process, whereby the reference list of all the included studies were scanned to discover potentially relevant studies, identified additional studies. No differentiation was made between studies obtained through the initial search, and those identified through snowballing. The snowball process also assisted to identify grey literature and information papers not published in peer reviewed journals.

### Study selection

Two reviewers independently assessed titles and abstracts. After scanning titles and abstracts, studies were identified for possible inclusion in the review using inclusion and exclusion criteria. The inclusion criteria were 1) the article states clear reasons for implementing specific interventions to improve health worker motivation and/or reduce medical migration; 2) the intervention(s) recommended by the article are linked to motivation; 3) article was in a developing country context. All papers selected for full-text retrieval were assessed using a quality checklist to identify if there was a clear statement of study aims and whether the methodology was appropriate. If either of these questions were not satisfied, the study was excluded from the review on the basis that it would not be useful in terms of understanding the study itself or comparing and collating the study within the review as a whole. If the individual study met both of the initial screening questions, it was assessed in further detail with a succession of follow up questions. Initial searches included literature reviews, opinion pieces and review articles. However, these were later excluded as the authors decided that using only primary studies would provide a stronger evidence base for analysing determinants of health worker motivation. Nevertheless, the results of this systematic review are compared to those of other non-systematic reviews in the discussion section.

### Data extraction and analysis

Due to the inclusion of mixed methodology papers, a narrative synthesis approach was adopted to summarise and synthesis findings. There is a lack of guidance on quality criteria applicable to mixed types of studies [19], so in order to appraise study quality each paper was evaluated by two reviewers. A data extraction form was adapted from Greenhalgh *et al *(2005) to summarise the research question, study design, robustness of methods, sample size, strength of findings and validity of conclusions [20]. The data were then reviewed and themes identified and analysed.

## Results

Electronic searching yielded 3,412 references. Papers were considered to merit scrutiny of the full article after their title and abstract had been considered. Of the articles identified as potentially relevant to the research question 108 were reviewed. After excluding studies which did not meet the stated inclusion criteria, or did not use primary data, twenty two remained, of which a further two were discarded as they did not meet criteria for quality. Consequently twenty papers met all the inclusion criteria. Of the included papers, eight used qualitative research methods, eight used quantitative research methods and four used a mixture of both qualitative and quantitative methodologies. The countries studied were from Africa (Benin, Cameroon, Ghana, Kenya, Malawi, Mali, Senegal, South Africa, Tanzania, Uganda, and Zimbabwe) and Asia (Bangladesh, Jordan, Georgia, Kazakhstan, Malaysia and Vietnam). For a brief description of the studies see additional file 1 (Description of studies).

### Themes identified within the included studies

Seven major themes regarding motivational factors were identified:

• Financial (in terms of salary or allowances)

• Career development (in regards to the possibility to specialise or be promoted)

• Continuing education (having the opportunity to take classes and attend seminars)

• Hospital infrastructure (the physical condition of the health facility, in papers often described as 'work environment')

• Resource availability (refers to equipment and medical supplies that are necessary for health workers to perform their job)

• Hospital management (refers to having a positive working relationship with the management with whom the health workers work and with)

• Personal recognition or appreciation (either from managers, colleagues of the community)

Other themes included fringe benefits (e.g. housing and transport allowances) [21,22], job security [23,24], personal safety [25], staff shortages [21,26] and social factors, such as effect on family life [21,22].

**Figure 1:**
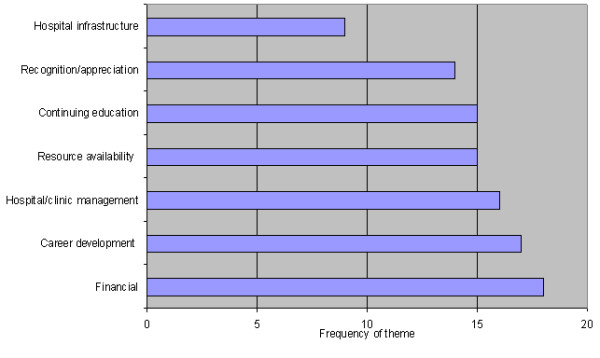
Occurrence of theme.

The number of articles that discussed a particular theme indicates how prevalent the theme is within the literature and is represented in Table 1 and Figure 1. There was not a requirement of a minimum number of themes to be included in the review, as these themes were only identified after the article had met the inclusion criteria.

**Table 1:** Major themes identified

Author (s)	Year	Financial	Career Development	Hospital or Clinic Management	Availability of resources	Continuing Education	Recognition or Appreciation	Hospital Infrastructure
Chomitz et al [38]	1998					**X**		
Sararaks & Jamaluddin [37]	1999	**X**	**X**	**X**	**X**	**X**	**X**	**X**
Bennett et al [28]	2000	**X**	**X**	**X**			**X**	
Stilwell [25]	2001	**X**			**X**		**X**	**X**
Awases et al [27]	2003	**X**	**X**	**X**	**X**	**X**		**X**
Dieleman et al [30]	2003	**X**	**X**	**X**		**X**	**X**	
Kyaddondo & Whyte [33]	2003	**X**		**X**	**X**	**X**	**X**	
Agyepong et al [21]	2004	**X**	**X**	**X**	**X**	**X**		
Franco et al [31]	2004	**X**	**X**	**X**			**X**	
Reid [23]	2004	**X**	**X**	**X**		**X**		
Chikanda [13]	2005	**X**	**X**	**X**	**X**	**X**		**X**
Penn-Kekana et al [36]	2005	**X**	**X**	**X**	**X**	**X**	**X**	**X**
Dielmen et al [29]	2006	**X**	**X**	**X**	**X**	**X**	**X**	
King & McInerney [32]	2006	**X**	**X**	**X**	**X**	**X**	**X**	**X**
Kotzee & ouper [22]	2006	**X**	**X**	**X**	**X**	**X**	**X**	**X**
Lephoko et al [39]	2006		**X**		**X**		**X**	
Manongi et al [26]	2006	**X**	**X**	**X**	**X**	**X**	**X**	**X**
Mathauer & Imhoff [35]	2006	**X**	**X**	**X**	**X**	**X**	**X**	**X**
Ssengooba et al [24]	2007	**X**	**X**	**X**	**X**		**X**	
Mangham & Hanson [34]	2008	**X**	**X**		**X**	**X**		

**Total number of studies**	**20**	**18 (90%)**	**17 (85%)**	**16 (80%)**	**15 (75%)**	**15 (75%)**	**14 (70%)**	**9 (45%)**

### Financial incentives

Almost all (90%) [13,21-37] of studies discussed the importance of financial incentives on health worker motivation. However, it was noted that financial incentives should be integrated with other incentives, particularly with regard to migration where it was concluded that financial incentives alone would not keep health workers from migrating [22,29-31,35]. Nevertheless, low salaries were found to be particularly de-motivating as health workers felt that their skills were not valued. Furthermore, they became overworked when taking a second job to supplement their income [30,31,33].

### Career development

Career development was identified in 85% of the studies [13,21-24,26-31,33,35,37]. Health workers were reluctant to work in rural areas as opportunities for career development were typically less than in urban areas [22]. The studies indicated that health workers take pride and are motivated when they feel they have the opportunity to progress. Job definition was also important, not only in terms of affecting general satisfaction and organizational commitment, but also for supervision and how staff assessed how they were getting along [28].

### Hospital or clinic management

The high frequency of this theme (80%) indicates the important role that management plays as a motivational factor [13,21-24,26-30,32]. Studies consistently provided opinions from health workers who stated that their supervisor's management and leadership skills were inadequate and this led to de-motivation of the workforce. Skilled managers have the ability to motivate their employees, however often in resource-poor institutions, management roles are assigned to staff who are not adequately trained. Effective managers are also responsible for lobbying on behalf of health workers and without their commitment factors affecting health worker motivation will not be identified or addressed.

### Education

Education and training opportunities have strong motivating effects [13,21-23,26,27,29,30,32-38]. Training enables workers to take on more demanding duties and to achieve personal goals of professional advancement [35] as well as allow them to cope better with the requirements of their job and was found to be especially important for young health professionals [23].

### Hospital infrastructure & resource availability

Hospital infrastructure and resource availability was a common theme [13,21-30,32-37] and lack of materials was an important de-motivator. Qualitative extracts show the need for basic drugs and equipment: *"we don't have a microscope or even a laboratory... we are only doing diagnosis and using our experience to decide. This is like playing a game of chance as you are not sure if you are treating malaria or typhoid or both...This is really discouraging" *[26]. Efforts must be made to ensure health workers are able to do their job utilizing their knowledge to the fullest and this should be an intrinsic component of any plan to increase retention. Hospital infrastructure and resource availability should be a principal consideration and patient care cannot be effective without the correct resources. Poor infrastructure does not inspire confidence from the health workers working there, nor from patients.

### Recognition/appreciation

Recognition and/or appreciation, either from managers, colleagues, or the community was a theme found in 70% studies [22,24-26,28-33,35-37,39]. In some articles, recognition by the employer and community was cited as being one of the most important motivating factors for health workers [29-31,33,35,37]. One health worker reported '*I feel that I do a good job. My boss appreciates me, but I do not know how. He does not say anything*'. Workers also reported that they were encouraged by getting results from their work, being useful to society and taking care of people [29]. In Tanzania although physical infrastructure and equipment were reported as being de-motivational factors, the need to feel valued and supported was much greater. It was also reported that to be trusted by the community was a crucial component for motivation [26].

## Discussion

The articles in this review explored motivational issues faced by health workers and made recommendations to improve health worker motivation. There were several common motivational themes identified and this was consistent with findings in opinion pieces and other non-systematic review articles, for instance that poor career paths and promotion opportunities lead to health workers feeling stuck and therefore more susceptible to the 'pull' factors of migration [40], and that improving working and living conditions maybe more effective than increasing wages to reduce migration flows [41]. Overwhelmingly the studies concluded that policies and packages of incentives are urgently needed to improve motivation and retention of health workers.

### Impact of Interventions to Improve Motivation

This review included studies on the effect of financial incentives on motivation [23,27]. In South Africa, rural allowances were found to have a limited effect on retaining workers [23]. The limited effect of financial allowances was also found in Cameroon and Zimbabwe where incentives have been perceived as unequally distributed between health workers. However, the fact that Uganda has the lowest level of those intending to migrate may be an indication that efforts to increase salary are working [27]. Most of the studies identified the need for financial and non-financial incentives, which is consistent with evidence from additional articles where was concluded that the best retention strategies combine both non-financial and financial incentives [14,42].

Health sector reforms had positive motivational effects in Bangladesh (through the securing of reliable, prompt payment of salaries) [24]. This is consistent with the effect of reforms in Kazakhstan (through better financial incentives and changed organisational relationships) [43] however, the potentially positive effect of Zimbabwean health sector reforms on motivation were undermined by poor communication and conflict with local cultures, resulting in workers perceiving the reforms as threatening their job security, salaries and career advancement [44]. In addition, it has been found that often reform programmes have focused on a limited number of channels, such as financial incentives to influence worker behaviour, and neglected less tangible incentives such as recognition and achievement [17].

### Differences across and within Cadres

In Georgia significant differences emerged between different cadres of worker with doctors rating financial rewards lower than non-clinical staff [28]. In Mali 'feeling responsible' received a significantly higher score by physicians compared to nurses and 'increase in salary' was significantly more motivating for nurses compared to physicians [29]. The incentive of specialist training in Indonesia was found to be enough to make urban doctors serve in rural locations [38]. In South Africa, nurses with children under 18 and in the age group 30–49 years were more likely to be considering going overseas than younger or older nurses [36].

Despite these findings insufficient evidence was found to draw conclusions on how motivational factors affect different cadres of health workers as most studies sampled health workers as a whole and results were not produced outlining how each cadre valued each motivational factor. Studies which did specifically sample one cadre of health worker had no comparisons to determine whether motivational factors were valued the same in another cadre.

### Evaluation of the Reviewed Studies

Studies were evaluated in order to identify flaws in study design and implementation as well as assess the wider significance of the results. A common theme reported in several studies was that interviewees found it difficult to express themselves and there were problems in how questions were understood with inconsistent interpretation of motivation related variables [28-30,35,38,39]. It is important for future motivational studies that variables are well understood by the field team and that they can clearly explain them, or that terms are clearly defined in any self-administered questionnaires. In addition, many of the studies were exploratory, with small sample sizes and it was not clear whether findings can be generalised, however the recurrence of the same themes in different countries among different cadres, albeit with differing emphasis, it can be assumed that the themes identified within the study are an accurate reflection of motivational issue faced by health workers.

### Study Limitations

A major limitation of this review is that only studies in English have been included, additionally databases of social sciences and humanities were not searched. There is also a lack of consistency in the detail of the study designs included in the review with different methodologies being used. Countries or regions are also affected by different issues and to varying extents these may affect the results, e.g. in Zimbabwe political and economic instability is likely to affect health workers more than in other countries. Approximately 60% of health workers in South Africa, Uganda and Zimbabwe reported they found it stressful to care for HIV patients [27]. Such important and distinct local or regional factors can compromise the utility of any systematic review.

## Conclusion

Strengthening health systems, especially at district level is critical to meeting the MDGs and human resources are essential to achieving this. High quality care cannot be provided unless issues of de-motivated staff are comprehensively addressed and more information is clearly needed to strengthen the evidence base for effective human resource strategies and policy decisions. Financial incentives, career development and management issues are core factors affecting motivation. It is clear that recognition is highly influential in health worker motivation; furthermore adequate supplies and appropriate infrastructure are factors that can significantly improve morale. Hence, financial incentives by themselves are not the appropriate response. Inconclusive evidence was found as to whether motivational factors are valued differently by different cadres and this needs to be explored further. Motivational factors are influenced by context and, therefore, it would be productive for future cross country research to use the same, or even standard, data collection tools, allowing more exploration of how context affects motivation, as well as to allow comparison of contextual factors. Additionally, motivational issues are transitional and where possible longitudinal research should be conducted to capture these changes. Consequently research around human resources must remain a priority.

## Competing interests

The authors declare that they have no competing interests.

## Authors' contributions

MW led on the initial article searching, identification, write up and analysis of the review. PB led on additional article identification, analysis, weighting of articles and write up. ST conceived of the original idea for the research, and helped design and supervised the research, assisting in the drafting, revision and finalisation. LW assisted in the supervision and revision of the research especially regarding the study methodology. DB and PD helped in revisions, identification of additional articles, evaluation of articles and final redrafting.

## Pre-publication history

The pre-publication history for this paper can be accessed here:



## Supplementary Material

Additional file 1 **Description of the studies.** This table provides a brief description of the studies included in the review. Click here for file
