# Supplementary material for: Motivation and retention of health workers in developing countries: a systematic review
Source: BMC Health Serv Res. 2008 Dec 4;8:247. doi: 10.1186/1472-6963-8-247 (PMC2612662; doi:10.1186/1472-6963-8-247)
Supplement: Additional file 1 — Description of the studies. This table provides a brief description of the studies included in the review. [file 1472-6963-8-247-S1.doc]

## Table 1 – Description of the studies

| **Author(s), year of publication** | **Country** | **Focus of study** | **Methodology** | **Participants** | **Main findings** |
| --- | --- | --- | --- | --- | --- |
| Chomitz *et al*  (1998) | Indonesia | To determine location choices made by graduating medical students and to determine preferences using hypothetical situations | *Quantitative*  Self-administered questionnaire (discrete choice) | Final year medical students | Incentives had a large impact on the willingness of graduates to volunteer for remote posts. Specialist training is a strong incentive and particularly so for males. |
| Sararaks & Jamaluddin (1999) | Malaysia | To determine de-motivating factors among government doctors | *Qualitative*  Self-administered open ended questionnaire | Doctors | Major de-motivating factors were remuneration and management related problems (recognition, relationships, training, workload and staffing) |
| Bennet *et al*  (2000) | Georgia | To compare motivational determinants between cadres and test associations | *Quantitative*  Individual worker questionnaire  Supervisor’s assessment of worker performance | Health workers & Supervisors | Issues concerning remuneration were overwhelming cited as being most critical followed by interventions to improve the work environment. |
| Stilwell (2001) | Zimbabwe | To improve understanding of factors which influence worker motivation | *Qualitative*  FGDs  Interviews | Nurses, Midwives, Managers &  Ministry of Health key informants | Key Motivators are sense of achievement, getting recognition and being valued for doing a great job. Key de-motivators are pay & working conditions. |
| Awases *et al*  (2003) | Cameroon, Ghana, Senegal, South Africa, Uganda, Zimbabwe | To determine the magnitude of migration of health professionals, the reasons for migrating and what can be done to retain health personnel. | *Quantitative*  Questionnaires  *Qualitative*  FGDs  In-depth interviews | Health workers, Policy makers, Representatives of private health sector/donors/NGOs & Community key informants. | Interviewees said they would return to their countries if attention is given to the major push factors. A strong desire was found for further training and international experience, this may benefit the migrant’s home country if they returned home after their period of study. An improved remuneration and a reward system would reduce the rate of out-migration in most of the countries studied. |
| Dieleman *et al*  (2003) | Vietnam | To determine what motivates and de-motivates health workers and their perceptions of Human Resource Management (HRM) tools | *Qualitative*  Semi-structured interviews  FGDs | Policy makers, Health workers & Community members | 5 motivating and 5 de-motivating factors were identified. Salaries and working conditions de-motivate workers and managers need to find appropriate HRM tools to motivate health workers to perform well. |
| Kyaddondo & Whyte  (2003) | Uganda | To examine the relation between health sector reform and health worker motivation | *Qualitative*  Interviews  FGDs  Observation | Health workers & Policy makers | Clear terms of employment and job descriptions facilitate willingness and commitment. Staff development enhances knowledge and skills and makes workers better able to perform, strengthening self-efficacy and motivating workers. |
| Agyepong *et al*  2004 | Ghana | Factors affecting health worker motivation and satisfaction as part of a ‘continuous quality improvement’ effort | *Quantitative*  Structured questionnaire | Health workers | De-motivating factors included low salaries, lack of essential equipment, delayed or perceived unfair promotions, difficulties with transport to work, inadequate staffing, housing, additional duty allowances, and inadequate training. |
| Franco *et al*  (2004) | Jordan & Georgia | To identify important motivational determinants and outcomes of worker motivation | *Qualitative*  Semi-structured interviews  *Quantitative*  Self administered questionnaire | Health workers, Key informants & Patients | All categories of determinants (e.g. self efficacy, pride, management, openness and job properties) had at least one significant impact on satisfaction, commitment, and cognitive motivation. |
| Reid  (2004) | South Africa | To evaluate the effect of the new rural allowance on the short term career choices of health professionals in rural areas | *Quantitative* (longitudinal cohort)  Self administered questionnaire | Health workers | The rural allowance had limited impact on retention and that equally important motivators were career development, job satisfaction and educational opportunities. |
| Chikanda  (2005) | Zimbabwe | Examine the magnitude of, and trends in, migration of nurses and midwives from Zimbabwe | *Quantitative*  Questionnaire | Nurses & Midwives | Reasons for intending to migrate were economic, political, professional and social. |
| Penn-Kekana *et al*  (2005) | South Africa | To assess the magnitude of maternal nursing staff dynamics | *Quantitative*  Self-administered questionnaires (discrete choice)  *Qualitative*  FGDs | Nurses, Midwives & Nursing managers | Good management and a well-equipped hospital were as influential over nurses’ choices as a 15% pay increase. A poor relationship with co-workers was associated with nurses’ intention to leave. |
| Dieleman *et al*  (2006) | Mali | To determine what motivates and de-motivates staff and the association for performance management activities | *Qualitative*  In-depth interviews  FGDs  *Quantitative*  Interview administered questionnaire | Health workers, Managers & Community members | Although salaries and incentives were important, recognition, responsibility and training were main motivators and gains in these would improve health worker performance. |
| Kotzee & Couper  (2006) | South Africa | To identify interventions to address the shortage of rural doctors | *Qualitative*  Semi-structured questionnaire | Doctors | Improving salary was one of the three most important factors in retaining rural doctors, followed by better accommodation and career progression. |
| Manongi *et al*  (2006) | Tanzania | To explore what motivates, satisfies and frustrates primary health care workers | *Qualitative*  FGDs | Health workers | Nine motivational themes were identified. Emphasis was placed supportive supervision and career development as well as infrastructure and equipment. To be trusted by the community was also mentioned as a crucial component for motivation. |
| Mathauer & Imhoff  (2006) | Kenya & Benin | Assess the impact of organizational determinants on the motivational process, specifically of HRM tools and non-financial incentives | *Qualitative*  Interview administered semi-structured interviews | Doctors, Nurses & Key informants in the Ministry of Health and at district level | Health workers are strongly guided by their professional conscience and aspects related to their professional ethos. |
| King & McInerney  (2007) | South Africa | To explore the reasons why registered nurses are resigning and describe hospital workplace experiences | *Qualitative*  Interview administered semi-structured questionnaire | Nurses | Five themes (including salary, working conditions and relationships) were identified and 22 sub-themes which participants considered contributory (to differing degrees) to their decision to resign. |
| Lephoko *et al*  (2007) | South Africa | To explore and describe organisational climate as a cause of job dissatisfaction among nursing staff. | *Quantitative*  Interview administered structured questionnaire | Nurses & Nurse managers | There is a relationship between organisational climate and job dissatisfaction. Findings could assist management teams in revising aspects and issues to positively influence the organisational climate within hospitals. |
| Ssengooba *et al*  (2007) | Uganda & Bangladesh | To examine the pathways through which selected health sector reforms & health worker responses to changes in their environment. | *Qualitative*  FGDs  Key informant interviews  *Quantitative*  Questionnaire | Health workers, Health care managers & Policy makers | The importance of contextual factor analysis in the design and implementation of reforms and recognition that the workforce contributes to the success of reforms. |
| Mangham & Hanson (2008) | Malawi | To determine the range and relative importance of factors influencing motivation of public sector nurses in Malawi. | *Qualitative*  In-depth interviews  *Quantitative*  Questionnaire (DCE) | Nurses | Pay was found to be the most important attribute, followed by further education and basic housing. Few socio-economic factors were found to influence employment preferences. |
